# Neuroprotektive Therapien bei idiopathischen, genetischen und atypischen Parkinson-Syndromen mit α-Synuklein – Pathologie

**DOI:** 10.1007/s00115-021-01220-y

**Published:** 2021-11-04

**Authors:** Johannes Levin, Georg Nübling, Armin Giese, Annette Janzen, Wolfgang Oertel

**Affiliations:** 1 Neurologische Klinik und Poliklinik, Ludwig-Maximilians-Universität München, München, Deutschland Marchioninistrasse 15, 83177; 2 Deutsches Zentrum für Neurodegenerative Erkrankungen e. V. (DZNE), München, Deutschland; 3 Munich Cluster for Systems Neurology (SyNergy), München, Deutschland; 4 Zentrum für Neuropathologie und Prionforschung, Ludwig-Maximilians-Universität München, München, Deutschland; 5 Klinik für Neurologie, Philipps-Universität Marburg, Baldingerstraße, 35043 Marburg, Deutschland

**Keywords:** Parkinson-Krankheit, Demenz vom Lewy-Körper-Typ, Multisystematrophie, Synukleinopathien, Krankheitsmodifzierende Therapie, Parkinson’s disease, Lewy body dementia, Multiple system atrophy, Synucleinopathies, Disease-modifying drugs

## Abstract

Kernpunkt der Klassifikation neurodegenerativer Erkrankungen ist der histopathologische Nachweis von Ablagerungen bestimmter Proteine im Gehirn. Hierbei unterscheiden sich die verschiedenen Krankheitsentitäten sowohl hinsichtlich der Art der nachweisbaren Proteine als auch hinsichtlich der Konfiguration und Lokalisation der entsprechenden Proteinaggregate. Gemeinsames Kernmerkmal der als Synukleinopathien zusammengefassten Erkrankungen sind Ablagerungen des Proteins α‑Synuklein (ASYN). Die bekanntesten Erkrankungen dieses Spektrums sind die Parkinson-Krankheit (PK) mit neuronalem Nachweis von Lewy-Körperchen, die Demenz vom Lewy-Körper-Typ (DLK) mit zusätzlichem Nachweis von β‑Amyloid-Ablagerungen sowie die seltene Multisystematrophie (MSA) mit glialem Nachweis sog. Papp-Lantos-Körperchen. Da neben der diagnostischen mittlerweile auch die zentrale pathophysiologische Bedeutung des ASYN erwiesen ist, fokussiert sich die Entwicklung neuer Therapien aktuell auf die Beeinflussung der toxischen Wirkung dieses Proteins. Die verschiedenen Therapiekonzepte lassen sich grob in sechs Gruppen zusammenfassen: 1. die Verringerung der ASYN-Expression (Antisense-Therapie), 2. die Verhinderung der Bildung toxischer ASYN-Aggregate (Antiaggregativa, Chelatoren), 3. das Auflösen bzw. die Beseitigung intra- oder extrazellulärer toxischer ASYN-Aggregate (aktive und passive Immuntherapie, Antiaggregativa), 4. die Verstärkung zellulärer Abräummechanismen (Autophagie, lysosomale Mikrophagie) zur Beseitigung toxischer Formen von α‑Synuklein, 5. die Modulation neuroinflammatorischer Prozesse sowie 6. neuroprotektive Strategien. In diesem Artikel fassen wir die aktuellen Therapieentwicklungen zusammen und geben einen Ausblick auf vielversprechende zukünftige Therapieansätze.

## Abstract (en)

The key aspect of the classification of neurodegenerative diseases is the histopathological detection of certain proteins in the brain. The various disease entities are distinguished with respect to the type of detected protein and with respect to the configuration and localization of the corresponding protein aggregates. Aggregates of alpha-synuclein (ASYN) are the defining hallmark of several neurodegenerative disorders termed synucleinopathies. The most well-known diseases in this spectrum are Parkinson’s disease (PD) with neuronal detection of Lewy bodies, dementia with Lewy bodies (DLB), with additional detection of beta-amyloid and multiple system atrophy (MSA), where ASYN aggregates are found in glia cells in the form of Papp-Lantos inclusions. ASYN has been identified as a key target for the development of therapeutic approaches to synucleinopathies given its central role in the pathophysiology of these diseases. Current treatment strategies can be roughly classified into six groups: 1) lowering ASYN expression (antisense therapy), 2) inhibition of formation of toxic ASYN aggregates (aggregation inhibitors, chelators), 3) dissolving or removal of intracellular or extracellular toxic AYSN aggregates (active and passive immunotherapy, aggregation inhibitors), 4) enhancement of cellular clearance mechanisms (autophagy, lysosomal microphagy) for removal of toxic forms of alpha-synuclein, 5) modulation of neuroinflammatory processes and 6) neuroprotective strategies. This article summarizes the current therapeutic approaches and sheds light on promising future treatment approaches.

## Hintergrund

Der Begriff Synukleinopathien fasst neurodegenerative Erkrankungen zusammen, deren gemeinsames Merkmal die pathologische Aggregation des Proteins α‑Synuklein (ASYN) zu histopathologisch nachweisbaren Ablagerungen in Nerven- und/oder Gliazellen ist. Als wichtigste Vertreter sind hier neben der Parkinson-Krankheit (PK) auch die Demenz vom Lewy-Körper-Typ (DLK) sowie die seltene Multisystematrophie (MSA) zu nennen, deren klinische Kernmerkmale in Tab. 1 zusammengefasst sind. Darüber hinaus zählen noch seltenere Krankheiten wie z. B. die isolierte autonome Funktionsstörung („pure autonomic failure“, PAF) zu den Synukleinopathien.

**Table 1:** 

Merkmale	Parkinson-Krankheit	Demenz vom Lewy-Körper-Typ	Multisystematrophie
Neuropathologie	Neuronale ASYN-Aggregate in Form von Lewy-Körpern beginnend im Bulbus olfaktorius bzw. Vaguskern mit Ausbreitung über Hirnstamm und Basalganglien	Neuronale ASYN-Aggregate in Form von Lewy-Körpern und Lewy-Neuriten mit Betonung in Neokortex und Hirnstamm	Zytoplasmatische ASYN-Aggregate in Oligodendrozyten (Papp-Lantos-Körperchen) mit Betonung in Kleinhirn, Basalganglien und der Pons
Prävalenz	Ca. 1 % der Bevölkerung jenseits des 60. Lebensjahres	Ca. 0,4 % der Bevölkerung jenseits des 65. Lebensjahres	3,4–4,9/100.000
Typisches Erkrankungsalter	50.–85. Lebensjahr, selten früher	50.–85. Lebensjahr	55.–60. Lebensjahr
Lebenserwartung	Gering reduziert	6–7 Jahre	3–10 Jahre
Motorische Symptome	Asymmetrisches hypokinetisch-rigides Syndrom mit Bradykinese, Rigor und/oder Ruhe-Tremor	Hypokinetisch-rigides Syndrom mit Beginn nach oder gleichzeitig mit dem Einsetzen kognitiver Defizite	Hypokinetisch-rigides Syndrom (MSA‑P, ca. 60 % der Fälle) ODER Zerebelläres Syndrom (MSA‑C, ca. 40 % der Fälle) mit Gangataxie, Dysarthrie, zerebellärer Okulomotorikstörung und Extremitätenataxie
Nichtmotorische Symptome	Frühsymptome: Hyposmie, Obstipation REM-Schlaf-Verhaltensstörung Dysautonomie Schlafstörungen Schmerzen Depression Kognitive Störung > 12 Monate nach Beginn der motorischen Symptome, i. d. R. spät im Krankheitsverlauf	Progrediente kognitive Störung mit vordringlicher Beeinträchtigung von Aufmerksamkeit, Exekutivfunktion und visuell-räumlichen Fähigkeiten Weitere Kernmerkmale: Fluktuationen in Wachsamkeit und Aufmerksamkeit Visuelle Halluzinationen REM-Schlaf-Verhaltensstörung	Obligatorisch mit ausgeprägter Dysautonomie mit Harninkontinenz, erektiler Dysfunktion, orthostatischer Hypotension REM-Schlaf-Verhaltensstörung

*ASYN* α‑Synuklein, *MSA* Multisystematrophie, *MSA-P* MSA Parkinson-Subtyp, *MSA-C* MSA-Cerebellärer Subtyp, *REM* „rapid eye movement“

Alpha-Synuklein ist ein aus 140 Aminosäuren bestehendes, primär ungefaltetes monomeres Protein. Die Existenz eines physiologischen α‑helikal gefalteten Tetramers (Molekulargewicht ca. 58 kDa) wird diskutiert [3]. Ursprünglich wurde ASYN als „nicht amyloider Anteil“ von Plaques bei der Alzheimer-Krankheit entdeckt. Im gesunden Gehirn kommt es hauptsächlich in präsynaptischen Nervenenden vor. Mehrere physiologische Funktionen des Proteins sind bekannt. Es beeinflusst aktivitätsabhängige Membrankanäle im Zusammenhang mit der Freisetzung von Dopamin, es spielt eine Rolle in der synaptischen Plastizität und es hat Chaperon-ähnliche Eigenschaften [38].

## Die Bedeutung von ASYN in der Pathogenese der Synukleinopathien

Der Nachweis einer zentralen Rolle des ASYN in der Pathogenese der Synukleinopathien erfolgte zunächst mit der Entdeckung mehrerer autosomal-dominanter Mutationen im für ASYN codierenden *SNCA*-Gen bei Familien mit erblicher PK [13]. Auch Duplikationen bzw. Triplikationen des nichtmutierten *SNCA*-Gens verursachen über eine Erhöhung der ASYN-Synthese eine autosomal-dominant vererbte PK, die einen sich früh manifestierenden und rasch progredienten Phänotyp mit in der Regel auch früh einsetzenden kognitiven Defiziten aufweist [7]. Der immunhistochemische Nachweis von ASYN in Lewy-Körpern in der Substantia nigra von Patienten mit idiopathischer (sporadischer) Parkinson-Krankheit legt nahe, dass ASYN auch in der Pathogenese nichterblicher Synukleinopathien eine zentrale Rolle spielt [34].

**Figure 1:**
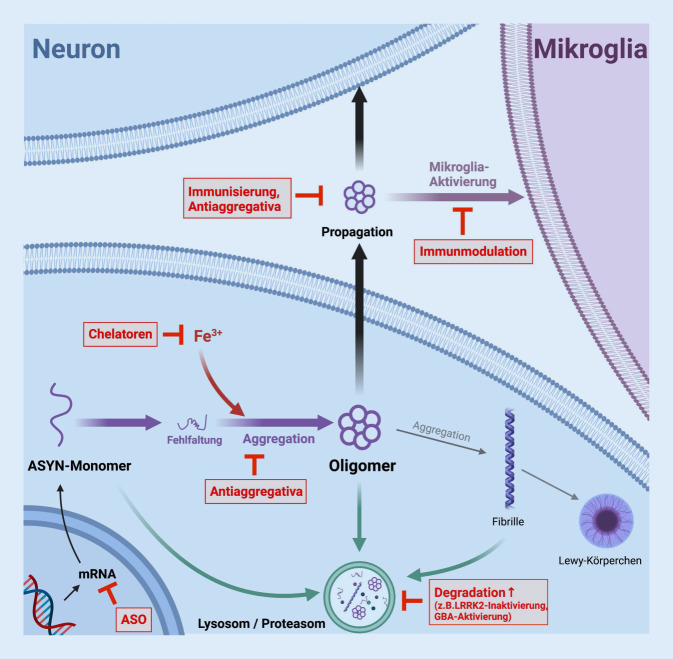

Pathophysiologisch kommt es bei den sporadischen ebenso wie bei den genetisch verursachten Synukleinopathien zur Fehlfaltung und Aggregation des ASYN. Die genauen Ursachen der erhöhten Aggregationsneigung des Proteins bei den sporadischen Erkrankungsformen sind noch unbekannt. Diskutiert werden verschiedene Mechanismen wie z. B. oxidativer Stress, regionale Veränderungen der Konzentration bestimmter Metallionen und posttranslationale Modifikationen [14]. Unabhängig von der genauen Ursache führen Aggregationsprozesse des in monomerer Form nichttoxischen ASYN zu zelltoxischen Oligomeren, die sich im Verlauf weiter zu Fibrillen organisieren und schließlich die mikroskopisch sichtbaren Lewy-Körper oder Papp-Lantos-Körperchen bilden. Die Bedeutung der Lewy-Körper wird kontrovers diskutiert – ob ihnen eine zellschädigende Rolle zukommt oder ob sie als Schutzmechanismus fungieren, um die ASYN-Belastung der Zelle zu mildern, ist noch nicht abschließend geklärt. Ebenso ist letztlich unklar, weshalb die Aggregation von ASYN bei den unterschiedlichen Krankheitsentitäten in unterschiedlichen Zellpopulationen und Gehirnregionen erfolgt. Biochemische Analysen in Post-mortem-Hirngewebe von Patienten mit PK, DLK und MSA haben nachgewiesen, dass sich deren ASYN-Aggregate in Zusammensetzung und 3‑dimensionaler Architektur voneinander unterscheiden [32]. Es scheint ähnlich wie bei Prionen auch bei ASYN-Aggregaten mehrere „strains“ zu geben ([33]; Abb. 1).

## Aktuelle Pharmakotherapie der Synukleinopathien

Alle aktuell bei Synukleinopathien angewandten Pharmakotherapien sind rein symptomatisch, verzögern also nicht die Krankheitsprogression. Die symptomatischen Behandlungsformen können nach Leitsymptomen gegliedert werden. Zur Verbesserung der motorischen Funktion beim Vorliegen eines hypokinetisch-rigiden Syndroms stehen zahlreiche Substanzen zur Verfügung, die die Dopaminkonzentration im synaptischen Spalt erhöhen oder dessen Wirksamkeit ersetzen oder verstärken. Unter Zuhilfenahme verschiedener Applikationsformen ist so bei der PK meist eine akzeptable bis gute Symptomkontrolle über 5 bis 8 Jahre möglich. Zu diesem Zeitpunkt wird die Therapie durch eine Zunahme von Wirkfluktuationen zunehmend komplexer. Neben der pharmakologischen Therapie stellt die tiefe Hirnstimulation des Nucleus subthalamicus (seltener auch der Pars interna des Globus pallidus) eine ergänzende Therapieoption dar. Unabdingbar sind besonders in fortgeschrittenen Krankheitsstadien supportive Therapieformen wie Physiotherapie, Ergotherapie und Logopädie zum Erhalt der Restfunktion.

In späteren Krankheitsstadien kommt es zur weiteren prionähnlichen Ausbreitung der ASYN-Pathologie und damit – in Abhängigkeit der betroffenen Hirnregionen – zu weiteren Einschränkungen, z. B. zu kognitiven Störungen. Eine Verbesserung der Kognition, z. B. bei der DLK, kann analog zur Alzheimer-Demenz vorübergehend über Cholinesterasehemmer erreicht werden, die Effekte sind jedoch moderat. Symptome einer autonomen Beteiligung, wie sie bei der MSA bereits früh, aber auch bei der PK im Verlauf häufig auftreten, sind in der Behandlung oft eine Herausforderung. Für Details zur symptomatischen Pharmakotherapie der Synukleinopathien verweisen wir auch auf andere Arbeiten, z. B. [11, 15]. Letztlich machen jedoch die Komplexität und auch die Grenzen der Pharmakotherapie der Synukleinopathien im Verlauf deutlich, dass dringend eine verlaufsmodifizierende Therapie benötigt wird.

## Natürlicher Erkrankungsverlauf und Überlegungen zur Therapieentwicklung

Zum jetzigen Zeitpunkt stehen keine verlaufsmodifizierenden Therapien für die Synukleinopathien zur Verfügung. Zahlreiche Studien mit Substanzen, die in vitro und in Tiermodellen den „Krankheitsprogress“ und/oder die Ausbreitung der ASYN-Pathologie hemmen konnten, zeigten in der Vergangenheit in Humanstudien keinen klinischen Effekt. Die Gründe hierfür sind unzureichend verstanden. Häufig wird – in Analogie zur Alzheimer-Krankheit (AK) – die Hypothese bemüht, dass Therapieeffekte nur bei sehr frühem Einsatz der Substanzen (z. B. vor der Entwicklung eines klinischen Phänotyps) eine Wirkung erzielen können, da bei späterem Einsatz die pathophysiologische Kaskade mit der Endstrecke der neuronalen Schädigung bereits zu weit vorangeschritten sei. Allerdings unterscheidet sich die Amyloidpathophysiologie deutlich von der Pathophysiologie der Synukleinopathien. So sind Amyloidablagerungen bei der AK bereits Jahrzehnte vor dem Eintreten einer relevanten Zellschädigung und eines entsprechenden klinischen Phänotyps nachweisbar und stellen letztlich das auslösende Agens der Kaskade dar, die langfristig den Zelluntergang herbeiführt [4]. Dies ist bei Synukleinopathien anders. ASYN ist hier eher analog zum Tau-Protein zu sehen, dessen Ablagerungen bei der AK im engen zeitlichen und örtlichen Zusammenhang mit dem klinischen Phänotyp stehen. Bei konsequenter Unterbindung der ASYN-Propagation sollten Therapieeffekte bei Synukleinopathien demzufolge auch in Krankheitsstadien mit relevantem (motorischem oder kognitivem) Phänotyp nachweisbar und somit auch objektivierbar sein.

Unabhängig von diesen Überlegungen ist perspektivisch die Behandlung von Patienten in einem möglichst frühen Stadium mit geringer oder fehlender (motorischer) Einschränkung anzustreben, um im Idealfall die Ausbreitung der ASYN-Pathologie auf verschiedene Hirnregionen zu verhindern. Aus diesem Grund ist die Entwicklung reliabler Biomarker zur frühzeitigen Detektion der Synukleinopathien im sehr frühen (präklinischen) bis frühen nonmotorischen (prodromalen) Erkrankungsstadien ein wachsendes Forschungsfeld. Unter den letzteren ist hier insbesondere die REM-Schlaf-Verhaltensstörung (RBD) als Kandidat für zukünftige neuroprotektive Therapiestudien zu nennen [11, 15, 22].

Die Prodromalphase der PK kann in mindestens 4 Stadien untergliedert werden

Histopathologisch kann die Prodromalphase der PK nach Braak in der überwiegenden Zahl der Parkinson-Patienten in mindestens 4 (maximal 6) Stadien untergliedert werden [5]. Im Stadium 0 beginnt die Erkrankung entweder im Bulbus olfactorius (führendes Symptom: Hyposmie) oder im gastrointestinalen System. Im Folgenden steigt die Erkrankung zum unteren Hirnstamm auf und schädigt hier insbesondere den Nucleus motoricus dorsalis des N. vagus (Stadium 1 – führendes Symptom: Obstipation). Der Krankheitsprozess erreicht im Verlauf den Locus coeruleus und die ihn umgebenden Kerne (Stadium 2 – führendes Symptom: REM-Schlaf-Verhaltensstörung). Im Rahmen der weiteren kaudorostralen Progression beginnt die Neurodegeneration des nigrostriatalen Systems, ohne jedoch motorische Symptome hervorzurufen (Stadium 3). Die klinische Diagnose PK nach etablierten Kriterien wird erst mit stärkerer Schädigung der dopaminergen Substantia nigra, Pars compacta im Übergang vom Stadium 3 zum Stadium 4 möglich.

Ähnliche klinische Beobachtungen sind für die DLK beschrieben. Bei dieser Erkrankung wird begleitend in der Mehrzahl der Fälle eine REM-Schlaf-Verhaltensstörung (RBD) beobachtet. Da diese bei isoliertem Auftreten oft und z. T. erst nach Jahren in eine DLK konvertiert, ist es wahrscheinlich, dass auch bei der DLK eine prodromale Phase existiert, bevor sich der kognitive Phänotyp manifestiert. Eine RBD verbunden mit einer leichten kognitiven Störung wird mittlerweile als prodromales Stadium der DLK angesehen [20].

## Krankheitsmodifizierende Therapieansätze

Wenngleich auch 2021 noch keine verlaufsmodifizierenden Therapien für Synukleinopathien zugelassen wurden, befinden sich mehrere vielversprechende Therapieansätze in der präklinischen Entwicklung oder bereits in der frühen, z. T. auch fortgeschrittenen klinischen Testung. Diese lassen sich orientierend in sechs Gruppen unterteilen (Abb. 1):

1. Verringerung der ASYN Expression (Antisense-Therapie),
2. Verhinderung der Bildung toxischer ASYN-Aggregate (Antiaggregativa, Chelatoren),
3. Auflösen/Abbau intra- oder extrazellulärer toxischer ASYN-Aggregate (aktive und passive Immuntherapie, Antiaggregativa),
4. Verstärkung zellulärer Abräummechanismen (Autophagie, Proteasom, Lysosom) zur Beseitigung toxischer Formen von ASYN,
5. Modulation neuroinflammatorischer Prozesse,
6. neuroprotektive Strategien.

Nachfolgend werden die einzelnen Therapiestrategien erläutert und Beispiele aktuell in der klinischen Prüfung befindlicher Substanzen im Detail beschrieben. Eine Übersicht derzeit laufender Studien (einschließlich deren Registernummern) ist in Tab. 2 zusammengestellt.

**Table 2:** 

Wirkmechanismus	Testsubstanz	Substanzklasse	Entwicklungsstand	Zielpopulation (*n*)	Registernummer (clinicaltrials.gov)
*1. Modulation der ASYN-Expression*					
Reduktion der *SNCA*-Expression	BIIB101	ASO	Phase 1	MSA (34)	NCT04165486
*2. Inhibition der ASYN-Aggregation/Auflösen von ASYN-Aggregaten*					
Direkte Inhibition der ASYN-Aggregation	Anle138b	SMC	Phase 1	HV (68)	NCT04208152
			Phase 1b	PK (24)	NCT04685265
	NPT200-11/UCB-0599	SMC	Phase 1	HV (55)	NCT02606682
			Phase 2	PK (300)	NCT04658186
Eisenchelatoren zur Aggregationsinhibition	PBT434 (AZT434)	SMC	Phase 1	HV (18)	ACTRN12618000541202
	NBMI	SMC	Phase 2	PSP und MSA (16)	NCT04184063
	Deferiprone	SMC	Phase 2	PK (372)	NCT02655315
*3. Immuntherapien*					
Passive Immunisierung	BAN0805/ABBV-0805	MAB	Phase 1, abgebrochen	PK (32)	NCT04127695
	Lu AF82422	MAB	Phase 1	HV (44)	NCT03611569
				PK (26)	
	TAK-341/MEDI1341	MAB	Phase 1	HV (48)	NCT03272165
			Phase 1	PK (26)	NCT04449484
	BIIB054 (cinpanemab)	MAB	Phase 2 abgebrochen 2021	PK (357)	NCT03318523
	PRX002/RO7046015 (prasinezumab)	MAB	Phase 2	PK (316)	NCT03100149
			Phase 2b (geplant)	PK (575)	NCT04777331
Aktive Immunisierung	UB-312	Polypeptid	Phase 1	PK (62)	NCT04075318
	AFFITOPE PD-01	Polypeptid	Phase 1	PK (32)	NCT01568099
	AFFITOPE PD-03		Phase 1	PK (36)	NCT02267434
	AFFITOPE PD-01 + PD-03		Phase 1	MSA (30)	NCT02270489
*4. Neuroinflammation*					
Toll-like-receptor-2-Antagonist	NPT520-34	SMC	Phase 1	HV (49)	NCT03954600
Depletion CD20-positiver Zellen	Rituximab	MAB	Phase 2	MSA (50)	NCT04004819
MAPK-p38-Inhibition	Neflamapimod	SMC	Phase 2	DLB (91)	NCT04001517
Hemmung der Myeloperoxidase	Verdiperstat (BHV-3241 or AZD 3241)	SMC	Phase 3	MSA (336)	NCT03952806
*5. Verstärkung zellulärer Mechanismen (Autophagie, lysosomale Mikrophagie) zur Beseitigung toxischer Formen von *α*-Synuklein*					
LRRK2-Inaktivierung durch Expressionsreduktion	BIIB094	ASO	Phase 1	PK (62)	NCT03976349
LRRK2-Inaktivierung	DNL151/BIIB122	SMC	Phase 1	PK (34)	NCT04056689
	DNL201	SMC	Phase 1	PK (29)	NCT03710707
Tyrosinkinaseinhibition zur Autophagieverstärkung	K0706/SCC-138	SMC	Phase 2	DLB (45)	NCT03996460
	Bosutinib	SMC	Phase 2	DLB (30)	NCT03888222
	Nilotinib	SMC	Phase 2	PK (75)	NCT02954978
			Phase 2	DLB (60)	NCT04002674
	FB-101/IST-102	SMC	Phase 1	HV (48)	NCT04165837
mTOR-Inhibition zur Autophagieverstärkung	Rapamycin	SMC	Phase 2 abgebrochen	MSA (56)	NCT03589976
Verstärkung lysosomaler Aktivität durch GCase-Stimulation	GZ/SAR402671 (venglustat)	SMC	Phase 2 gestoppt	PK mit GBA-Mutation (270)	NCT02906020
	Ambroxol	SMC	Phase 2	PKD (75)	NCT02914366
			Phase 1/2	DLB (15)	NCT04405596
			Phase 2	DLB (172)	NCT04588285
*6. Neuroprotektion*					
Inflammationsmodulation und Exkretion trophischer Faktoren	Mesenchymale Stammzellen	–	Phase 1	MSA (8)	NCT04495582
			Phase 1	MSA (9)	NCT03265444
			Phase 1	MSA (30)	NCT02315027
Inhibition des Apoptosesignalwegs via Fas-assoziiertem Faktor 1	KM-819	SMC	Phase 1	HV (88)	NCT03022799
„Cerebral dopamine neurotrophic factor“	CDNF	Protein	Phase 1/2	PK (17)	NCT03295786
Antioxidativer Effekt	Inosin 5′-Monophosphat	SMC	Phase 2	MSA (43)	NCT03403309
Inflammationsmodulation, Neuroprotektion	Exenatide (Glukagon-like-peptid-1-rezeptor-agonist)	SMC	Phase 3	PK (300)	NCT04232969
			Phase 2	MSA (50)	NCT04431713
			Phase 2	PK (60)	NCT04305002
	Exenatide („sustained release“)		Phase 2	PK (99)	NCT04269642
	NLY01 („pegylated Exenatide“)		Phase 2	PK (240)	NCT04154072

*ASO* Antisense-Oligonukleotid, *DLB* „dementia with Lewy bodies“, *GBA* Glukozerebrosidase, *LRRK2* „leucine-rich repeat kinase 2“,* MAB* monoklonaler Antikörper, *MSA* Multisystematrophie,* mTOR *„mammalian target of rapamycin“, *PK *Parkinson-Krankheit, *SMC* „small molecular compound“, *HV* healthy volunteers, *PKD* Parkinson-Krankheit mit Demenz, *PSP* Progressive supranukleäre Blickparese

### Antisense-Oligonukleotid-Therapie – Reduktion der Synthese von ASYN

Das Prinzip der Antisense-Oligonukleotid(ASO)-Therapie beruht auf der Abnahme der Genexpression des für ASYN codierenden *SNCA*-Gens auf RNA-Ebene und der damit verbundenen Reduktion der Proteinsynthese. Grundlage dieser Therapiestrategie ist die Beobachtung, dass eine Ausschaltung des *SNCA*-Gens in adulten Wildtypmäusen mit einem sehr milden Phänotyp einhergeht [12]. Antisense-Oligonukleotide erlauben es, die Genexpression auf verschiedenen Wegen zu verändern. Sie können die Translation blockieren, die Stabilität der mRNA verändern, das Splicing modifizieren oder Abbaumechanismen der RNA aktivieren. Darüber hinaus gibt es weitere Möglichkeiten, die Genexpression zu beeinflussen, z. B. mittels RNA-Interferenz, Ribozymen oder Deoxyribozymen oder RNA-Aptamere.

In tierexperimentellen Studien führte intrathekal appliziertes ASYN-ASO in ASYN-transgenen Mäusen zu einer deutlichen Reduktion der α‑Synuklein-Synthese [1]. Weitere präklinische Untersuchungen konnten zeigen, dass ASO den striatalen Dopamingehalt in einem Mausmodell der PK verbessern und in Primaten die Synukleinkonzentration im Liquor senken kann [8].

Basierend auf diesen präklinischen Befunden wurde bereits im Juli 2020 die erste klinische placebokontrollierte Phase-1-Studie mit monatlicher intrathekaler Gabe des ASYN-ASOs BIIB 101 bei 34 MSA-Patienten initiiert.

### Aggregationsinhibitoren: „small molecular compounds“ und Chelatoren

Inhibitoren der ASYN-Aggregation verfolgen das Ziel, die Entstehung pathophysiologisch relevanter toxischer ASYN-Oligomere zu verhindern und auf diesem Wege auch eine interzelluläre Ausbreitung der ASYN-Pathologie zu unterbinden. Bei den Aggregationsinhibitoren handelt es sich meist um sog. „small molecular compounds“, also chemisch synthetisierte Substanzen, die in Hochdurchsatzverfahren hinsichtlich ihrer antiaggregatorischen Eigenschaften in vitro untersucht und nach weiteren Gesichtspunkten wie Liquorgängigkeit oder Löslichkeit für in-vivo-Versuche selektiert werden. In anderen Fällen werden potenzielle Aggregationsinhibitoren z. B. aus Pflanzen oder anderen biologischen Quellen isoliert. Wenngleich auf diesen Wegen bereits eine Vielzahl potenzieller Aggregationsinhibitoren identifiziert wurde [6, 23, 43], finden aufgrund verschiedener Probleme wie Toxizität oder Gewebegängigkeit nur wenige Substanzen den Weg in die klinische Studienphase.

Aktuell rekrutiert eine im Dezember 2020 begonnene Phase-2-Studie zur Erprobung von UCB-0599 (vormals NPT200-11) 300 PK-Patienten im frühen Krankheitsstadium, nachdem eine vorangegangene Phase-1b-Studie die Verträglichkeit der Substanz bei PK-Patienten demonstrieren konnte. Die Wirkung von UCB-0599 zielt darauf ab, die Bildung membranassoziierter ASYN-Dimere zu unterbinden, die als Nukleationskerne größerer Aggregate fungieren. Hierzu bindet UCB-0599 selektiv an das N‑terminale Motiv der Aminosäuren 96–102 des Proteins. In vitro konnte eine Reduktion der ASYN-Membranbindung und -Oligomerbildung demonstriert werden [45]. Im Mausmodell reduzierte die Substanz die ASYN-Pathologie und verbesserte die motorische Funktion [28].

Ebenfalls in einer frühen Phase der klinischen Erprobung befindet sich das Diphenyl-Pyrazol Anle138b (aktuell Phase-1b-Studie bei PK). Eine im vergangenen Jahr durchgeführte Studie an gesunden Probanden konnte erfolgreich abgeschlossen werden. In den gesunden Freiwilligen wurden bei hoher Sicherheit und Verträglichkeit Gewebsspiegel deutlich jenseits der in Tierversuchen ermittelten Wirksamkeitsschwelle erreicht. Anle138b bindet selektiv an oligomerspezifische strukturelle Epitope und inhibiert Wechselwirkungen zwischen SNCA-Monomeren, die zur Stabilisierung pathologischer Oligomere notwendig sind. Daher hat Anle138b antiaggregative bzw. oligomerauflösende Eigenschaften in vitro und führt in verschiedenen Mausmodellen zu einer Verminderung der ASYN-Pathologie und Verbesserung des Phänotyps. Hervorzuheben ist dabei, dass Anle138b sowohl im PK-Tiermodell als auch im MSA-Tiermodell günstige Effekte zeigt und auch dann noch wirksam ist, wenn die Tiere bereits einen Phänotyp entwickelt haben [16, 43, 44].

Eisenchelatoren inhibieren die aggregationsfördernde Wirkung von Eisenionen

Eine weitere Herangehensweise zur indirekten Inhibition der ASYN-Aggregation liegt in der Modifikation aggregationsfördernder Faktoren. Hier sind insbesondere Eisenionen im Fokus der Therapieentwicklung. Es konnte gezeigt werden, dass die Substantia nigra bei PK-Patienten einen erhöhten Eisengehalt aufweist [9]. Zudem wirken insbesondere dreiwertige Eisenionen stark aggregationsfördernd [14]. Dementsprechend wurden mehrere z. T. bereits für andere Erkrankungen in klinischer Anwendung befindliche Eisenchelatoren hinsichtlich ihres therapeutischen Potenzials bei Synukleinopathien untersucht. Derzeit befinden sich drei Substanzen in der klinischen Entwicklungsphase. Für Deferiprone konnte in zwei Phase-2-Studien eine Verminderung des zerebralen Eisengehalts nachgewiesen werden, wobei nur eine der Studien einen Effekt auf den Progress motorischer Symptome zeigen konnte. Eine Phase-3-Studie steht kurz vor dem Abschluss. Mit dem Ziel, potenzielle negative Effekte einer starken Eisenreduktion zu minimieren, wurde der niedrig-affine Chelator PBT434 entwickelt. Dieser zeigte ebenfalls antiaggregative und neuroprotektive Eigenschaften in vitro und tierexperimentell in vivo sowie eine gute Verträglichkeit in einer zwischenzeitlich abgeschlossenen Phase-1-Studie [10, 35].

### Beseitigung extrazellulärer ASYN-Aggregate: Immuntherapien

Die Rationale der Immuntherapie gründet sich auf der Hypothese, dass die Ausbreitung von ASYN im Sinne eines prion-ähnlichen Mechanismus stattfindet. Danach erfolgt der Transport toxischer ASYN-Aggregate aus dem intrazellulären in den extrazellulären Raum. Von hier aus werden diese ASYN-Aggregate von benachbarten Zellen aufgenommen, um hier als Anlagerungskern für monomeres endogenes ASYN zu dienen [17]. Nach dieser Hypothese sollte die extrazelluläre Elimination toxischer ASYN-Aggregate die Ausbreitung der ASYN-Pathologie unterbinden und das klinische Voranschreiten der Erkrankung verlangsamen. Eine besondere Herausforderung für die passive Immuntherapie stellt hierbei die Überwindung der Blut-Hirn-Schranke dar, da geschätzt wird, dass maximal 2 % der infundierten Antikörper das Zentralnervensystem (ZNS) erreichen.

#### Aktive Immuntherapie

Erste Pilotstudien zur aktiven Immunisierung gegen ASYN-Epitope dienten dem Nachweis der Verträglichkeit sowie des Immunisierungserfolgs. Die Substanz PD01A wurde in einer Phase-1-Studie bei PK erprobt und zeigte, dass bei der Mehrzahl der Probanden eine Immunisierung gegen ASYN nachgewiesen werden konnte [40]. Eine weitere Phase-1-Studie an frühen Parkinson-Patienten, diesmal mit randomisiertem kontrolliertem Studienplan setzte eine andere Peptidmischung (PD03A) für die aktive Immunisierung gegen ASYN ein. Zudem wurde in einer kombinierten Studie entweder PD01A oder PD03A an 30 MSA-Patienten erprobt [21]. Eine Phase-2-Studie wurde 2020 angekündigt, bislang aber noch nicht umgesetzt. Alle drei Studien konnten die Entwicklung ASYN-spezifischer Antikörper im Serum nachweisen, im Liquor waren diese nur bei PD01A detektierbar [26]. Ein Effekt auf klinische Symptome wurde nicht beschrieben, da die o. g. Studien diese lediglich als explorative Endpunkte mitführten. Aktuell laufend ist eine weitere Phase-1-Studie an insgesamt 62 gesunden Probanden und PK-Patienten mit dem Vakzin UB-312. Ergebnisse werden im kommenden Jahr erwartet.

#### Passive Immuntherapie

Mehrere gegen ASYN gerichtete Antikörper sind derzeit in klinischer Entwicklung. Wichtige klinische Meilensteine, namentlich ein Reduktionsnachweis des freien ASYN im Serum und der Nachweis der Antikörper im Liquor, konnten – bei guter Verträglichkeit – die Antikörper Prasinezumab und Cinpanemab erreichen. In der Entwicklung am weitesten fortgeschritten ist der monoklonale Immunglobulin(Ig)G1-Antikörper Prasinezumab. Er befindet sich aktuell in einer Phase-2b-Studie (PADOVA) sowie einer offenen Verlängerungsphase („open label extension“) der vorangegangenen PASADENA-Studie (Phase 2) mit 316 Patienten. Diese untersuchte den Effekt von Prasinezumab auf vorwiegend motorische Endpunkte bei monatlicher intravenöser Gabe in zwei Dosisstufen, 1500 mg oder 4500 mg. Als Besonderheit wurden hier sekundäre Endpunkte in Form motorischer und nichtmotorischer Tests aufgenommen, die die Probanden regelmäßig über ihr Mobiltelefon ausführen sollten. Wenngleich der primäre Endpunkt einer verzögerten Progression motorischer Symptome im MDS-UPDRS (Teil 3) verfehlt wurde, wurden positive Effekte auf mehrere sekundäre Endpunkte zum Anlass genommen, die Entwicklung in Form der Phase-2b-Studie (PADOVA) bei PK-Patienten fortzusetzen.

Eine Phase-2-Studie mit Cinpanemab wurde vorzeitig abgebrochen, da Endpunkte der Zwischenevaluation nicht erreicht wurden. Weitere Antikörper in der frühen klinischen Testung an PK-Patienten und gesunden Probanden sind MEDI1341 und Lu AF82422.

### Verstärkung der zellulären ASYN-Clearance

Der Abbau von ASYN erfolgt über verschiedene Mechanismen:

- eine Chaperon-vermittelte Autophagie,
- eine lysosomenvermittelte Mikroautophagie,
- den Ubiquitin-Proteasom-Weg und
- die Bildung von Aggresomen [36].

Genetische Befunde deuten darauf hin, dass ein verminderter Abbau von ASYN zur Pathogenese von Synukleinopathien beiträgt. So führen ursprünglich bei der familiären PK beschriebene Funktionsgewinnmutationen in dem Gen, das für die „leucin-rich repeat-kinase 2“ (*LRRK2* = *PARK8*) codiert, zu vermindertem ASYN-Abbau [37]. Inzwischen wurden *LRRK2*-Mutationen auch in einigen pathologisch nachgewiesenen MSA-Fällen gefunden [29]. Darüber hinaus wurden Varianten der Glukozerebrosidase (GBA), die die Autophagie-Lysosomen-Clearance von ASYN beeinflussen, sowohl mit der PK als auch mit der MSA in Verbindung gebracht.

Zusammengenommen deuten diese genetischen Befunde auf eine Assoziation hin zwischen dem Auftreten von Synukleinopathien und Veränderungen der ASYN-Degradation hin. Daraus leitet sich die Strategie ab, die Clearance von ASYN-Aggregaten und/oder den ASYN-Stoffwechsel zu verbessern. Mehrere entsprechende Ansätze befinden sich in einem frühen Stadium der klinischen Entwicklung, darunter die LRRK2-Inaktivierung und die Verringerung der LRRK2-Genexpression durch ein weiteres ASO (BIIB 094), welches am *LRRK2*-Gen ansetzt. Auch die c‑Abl-Hemmung, die mTOR(„mammalian target of rapamycin“)-Hemmung und die Steigerung der lysosomalen Aktivität durch Stimulierung von GBA werden verfolgt. Die Inaktivierung oder Reduktion von LRRK2 zeigte in ASYN-basierten Tiermodellen uneinheitliche Effekte [41], ebenso wie die Hemmung von mTOR und c‑Abl. Im Gegensatz dazu zeigte die Stimulation von GBA in verschiedenen Synukleinopathie-Modellen konsistentere Wirkungen [30, 31].

### Neuroinflammation

Weitere Strategien zielen auf Mechanismen der Krankheitsprogression ab, die der ASYN-Aggregation nachgelagert sind, wie z. B. die Neuroinflammation. Neuroinflammation in Form glialer Aktivierung kann bei zahlreichen neurodegenerativen Erkrankungen beobachtet werden und fällt bei der MSA besonders deutlich aus. Eine Rolle der neuroinflammatorischen Reaktion in der voranschreitenden Pathophysiologie insbesondere der MSA ist sehr wahrscheinlich. Dementsprechend konzentrieren sich Studien zur Immunmodulation auf diese Krankheitsentität. Zu den Strategien, die das Fortschreiten der Krankheit über die Modulation der Neuroinflammation beeinflussen, gehören die Hemmung der Mikroglia oder die Verringerung der allgemeinen Immunantwort durch Hemmung der Myeloperoxidase, Toll-like-Rezeptor-2-Antagonisten oder die Verarmung CD20-positiver Zellen. Aktuell sind mehrere Substanzen in der klinischen Studienphase angekommen, wobei der Myeloperoxidaseinhibitor Verdiperstat mit einer laufenden Phase-3-Studie in der Entwicklung am weitesten vorangeschritten ist. Zur Erprobung von Rituximab rekrutiert eine Phase-2-Studie aktuell MSA-Patienten, und für NTP520-34, einen Antagonisten am Toll-like-Rezeptor 2, ist eine Phase-1-Studie an gesunden Probanden abgeschlossen.

### Neuroprotektion

In der Vergangenheit sind zahlreiche im weiteren Sinne potenziell neuroprotektive Substanzen bei der PK getestet worden. Die Auswahl der Substanzen beruhte auf Ergebnissen aus der tierexperimentellen sowie der epidemiologischen Forschung. Zu ersteren gehört der Wachstumsfaktor Neuturin, der mittels eines Adeno-assoziierten Virusvektors in das Gehirn injiziert wurde. Weitere Substanzen sind Koenzym Q 10 und Kreatinin, trophische Faktoren wie „glial cell-derived neurotrophic factor“ (GDNF), Inosin, Pioglitazon sowie der Dopaminagonist Pramipexol. Für keinen dieser Ansätze konnte ein verlaufsmodifizierender Effekt nachgewiesen werden. Weitere Strategien, das neuronale Überleben zu verbessern, beinhalten die Transplantation mesenchymaler Stammzellen, die nachweislich trophische Faktoren ausscheiden und die Neuroinflammation modulieren.

Epidemiologische Studien zeigten eine inverse Korrelation zwischen der Einnahme bestimmter Genussmittel und dem PK-Risiko. Besonders hervorzuheben sind hier Koffein und Nikotin. Auch für das Medikament Isradipin, einen Kalziumkanalblocker mit relativ hoher Affinität für Cav1.3 Kanäle, existieren entsprechende epidemiologische Hinweise. Aufgrund dieser epidemiologischen Studien sind große Untersuchungen mit Nikotin, Koffein und Isradipin durchgeführt worden. Eine placebokontrollierte randomisierte Studie mit additiver Koffeingabe verzögerte die Krankheitsprogression nicht [27]. Mehrere randomisiert-kontrollierte Studien zur Wirksamkeit von Nikotin zeigten ebenfalls negative Ergebnisse, einschließlich der größten Studie zur Wirksamkeit transdermalen Nikotins bei De-novo-PK-Patienten über ein Jahr, deren vorläufige Ergebnisse als Abstract publiziert wurden [19, 39, 42]. Ähnliche Ergebnisse ergaben die Studien mit Isradipin [25].

Weitere epidemiologische Daten wiesen darauf hin, dass erhöhte Serumuratspiegel einerseits mit einer Reduktion des PK-Risikos, andererseits bei PK-Patienten im frühen Stadium mit einer langsameren Krankheitsprogression assoziiert sind. Der Uratvorläufer Inosin kann die Uratspiegel erhöhen, weswegen auch er klinisch getestet wurde. Die Behandlung für 8 bis 24 Monate zeigte bei guter Verträglichkeit keine Veränderung der motorischen Symptome [24].

Die vielversprechendste aktuell in der klinischen Testung befindliche neuroprotektive Substanz ist der Glukagon-like-peptid-1(GLP-1)-Rezeptor-Agonist Exenatide. Die Stimulation des von dopaminergen Neuronen exprimierten Rezeptors hatte einen neuroprotektiven Effekt im MPTP(1-Methyl-4-phenyl‑1,2,3,6-tetrahydropyridin)-Mausmodell [18]. Hier sind derzeit fünf Phase-2/3-Studien mit einer avisierten Patientenzahl von 750 aktiv, die die Substanz u. a. in einer Slow-release-Formulierung und in pegylierter Form unter der Kennung NLY01 untersuchen. Eine 2017 publizierte Phase-2-Studie zeigte positive Effekte auf motorische Symptome [2], nachdem die Substanz zuvor neuroprotektive Effekte in verschiedenen Tiermodellen gezeigt hatte.

Zusammenfassend muss festgestellt werden, dass die Entwicklung von Therapeutika basierend auf epidemiologischen Daten bzw. der Beobachtung neuroprotektiver Effekte in Tier- oder Zellmodellen bislang enttäuschend verlief. Analog zu anderen Therapiekonzepten ist es auch hier möglich, dass der Einsatz der Substanzen zu spät im Krankheitsprogress erfolgt ist. Ebenso denkbar ist jedoch, dass die epidemiologischen Beobachtungen auf noch nicht identifizierte Confounder hinweisen.

## Fazit für die Praxis

- Trotz intensiver Bemühung stehen zum heutigen Tag noch keine verlaufsmodifizierenden Therapien der Synukleinopathien zur Verfügung. Allerdings waren die Forschungs- und Entwicklungsaktivität noch nie so hoch wie aktuell.
- Basierend auf dem klaren Zusammenhang zwischen pathologischer α‑Synuklein(ASYN)-Aggregation und der Diagnose und dem klinischen Phänotyp der Synukleinopathien haben die Autoren die große Hoffnung, dass die neuen molekular gut begründeten Ansätze bessere Erfolgschancen mit sich bringen und sind der Meinung, dass gezielt auf ASYN ausgerichtete Therapieansätze besonders vielversprechend sind.
- Die kürzlich in den USA erfolgte Zulassung des Antikörpers Aducanumab macht diese Fortschritte bei der Therapieentwicklung mit pathologischen Proteinaggregaten als Ziel sichtbar und weckt die Hoffnung, dass eine vergleichbare Dynamik auch auf dem Feld der Synukleinopathien möglich ist. Möglicherweise werden schon bald erste Durchbrüche auf dem Weg zu krankheitsmodifizierenden Therapien von Synukleinopathien erzielt.
